# Let Nothing Be Wasted

**Published:** 1875-01

**Authors:** 


Let Nothing be Wasted.—Stories are told of battle-fields being plundered for the sake of the decaying bones of the soldiers who had fallen. Bones, new and old, whenever they can be picked up, are put to a variety of uses. The fresher kinds of shank-bones serve for making the handles of knives, forks, and tooth-brushes. From some, gelatine is extracted. When not serviceable for these purposes, they are crushed into powder for manure. Farmers buy this bone-dust in large quantities for fertilizing their fields. It is used in immense quantities in England and elsewhere. Researches for the material of bone-dust are carried on upon a large scale in the ancient cemeteries and pyramids of Egypt. Long ago, when the people of that country mummified the bodies of their relations, and stowed them ceremoniously away in caverns, they were not aware that they were only preserving them for manure for English farms. Fancy mutton fattened on ancient Egyptians! At Sakhara, we saw nine camels pacing down from the mummy pits to the bank of the river, laden with nets, in which were femora, tibiæ, and other bony pits of the human form, some two hundred weight in each net on each side of the camel. Among the pits were people busily engaged in searching out, sifting, and sorting the bones which almost crust the ground. On inquiry we learned that the cargoes with which the camels were laden would be sent down to Alexandria, and thence be shipped to English manure manufacturers. They make excellent manure, particularly for swedes and other turnips. The trade is brisk, and has been going on for years, and may go on for many more. It is a strange fate—to preserve one's skeleton for thousands of years in order that there may be fine Southdowns and Cheviots in a distant land! But Egypt is always a place of wonders.

Nothing seems to be so thoroughly used up as old clothes. The buying and selling of cast-off apparel is a great business in London. Usually the worn garments are freshened up by dyestuffs, pressed and otherwise doctored for the market. The process of dressing them is called clobbering, and this in itself is a business. The better class of old-dress coats, when nicely clobbered, have a respectable appearance, and sell readily to persons whose small means would never enable them to purchase costly new garments. Coats and other woolen garments which have done good service are exported to Ireland and Holland, where you may see them in great quantities for sale at the fairs and markets. As regards the sale of second-hand ladies' dresses, the trade is everywhere on the increase. Silks, laces, shawls, frills, and all sorts of frippery are purchased by dealers, whose names are seen in advertisements, and are retailed by them on a very comprehensive scale or exported. The chief customers for the used, though, in many cases, elegant dresses, are ladies who aspire to a showy exterior.

However woolen garments may be disposed of time after time, they are at length no longer passable, and then comes a total revolution in their character; the buttons are taken off, the linings torn out, and what remains of the fabric is ground by machinery into "devil's dust." This is the first step in what may be called the resurrection in old clothes. When a coat will not so much as hang together to dress up a scarecrow, it will still make very good shoddy, as the devil's dust is politely named. The meaning of this is that the garment is torn up by toothed wheels into a condition of loose fibers, which, on being properly sifted, are mixed with fresh wool, carded, spun, and woven into cloth. There is a triumph of art! The shoddy, or mungo, as it is sometimes called, after being fit for the dungheap, is incorporated with what appears to be exceedingly beautiful cloth, and is again proudly exhibited as Sunday clothes on the backs of thousands of wearers. The thing seems ridiculous, if not a bit of a cheat; but let us not be too hard on shoddy. There is not a sufficiency of fresh wool for all the world. And as woolen goods are in ever-growing demand, what better can be suggested than that the elastic fibers of the old garments should be wrought up into an article agreeable to the eye, and productive ot bodily comfort? All hail to the value and virtues of shoddy! He was a great man, who thought out that marvelous invention.

